# Transcutaneous Auricular Vagus Nerve Stimulation Promotes White Matter Repair and Improves Dysphagia Symptoms in Cerebral Ischemia Model Rats

**DOI:** 10.3389/fnbeh.2022.811419

**Published:** 2022-04-04

**Authors:** Lu Long, Qianwen Zang, Gongwei Jia, Meng Fan, Liping Zhang, Yingqiang Qi, Yilin Liu, Lehua Yu, Sanrong Wang

**Affiliations:** ^1^Department of Rehabilitation Medicine, The Second Affiliated Hospital of Chongqing Medical University, Chongqing, China; ^2^Department of Traditional Chinese Medicine, Weinan Central Hospital, Weinan, China; ^3^Center of Electron Microscope, Institute of Life Science of Chongqing Medical University, Chongqing, China

**Keywords:** ta-VNS, white matter, dysphagia after stroke, angiogenesis, inflammation

## Abstract

**Background:**

Clinical and animal studies have shown that transcutaneous auricular vagus nerve stimulation (ta-VNS) exerts neuroprotection following cerebral ischemia. Studies have revealed that white matter damage after ischemia is related to swallowing defects, and the degree of white matter damage is related to the severity of dysphagia. However, the effect of ta-VNS on dysphagia symptoms and white matter damage in dysphagic animals after an ischemic stroke has not been investigated.

**Methods:**

Middle cerebral artery occlusion (MCAO) rats were randomly divided into the sham, control and vagus nerve stimulation (VNS) group, which subsequently received ta-VNS for 3 weeks. The swallowing reflex was measured once weekly by electromyography (EMG). White matter remyelination, volume, angiogenesis and the inflammatory response in the white matter were assessed by electron microscopy, immunohistochemistry, stereology, enzyme-linked immunosorbent assay (ELISA) and Western blotting.

**Results:**

ta-VNS significantly increased the number of swallows within 20 s and reduced the onset latency to the first swallow. ta-VNS significantly improved remyelination but did not alleviate white matter shrinkage after MCAO. Stereology revealed that ta-VNS significantly increased the density of capillaries and increased vascular endothelial growth factor (VEGF) and basic fibroblast growth factor (FGF2) expression in the white matter. ta-VNS significantly alleviated the increase inTLR4, MyD88, phosphorylated MAPK and NF-κB protein levels and suppressed the expression of the proinflammatory factors IL-1β and TNF-α.

**Conclusion:**

These results indicated ta-VNS slightly improved dysphagia symptoms after ischemic stroke, possibly by increasing remyelination, inducing angiogenesis, and inhibiting the inflammatory response in the white matter of cerebral ischaemia model rats, implying that ta-VNS may be an effective therapeutic strategy for the treatment of dysphagia after ischemic stroke.

## Introduction

Ischemic stroke, a very common health problem worldwide, leads to a decline in quality of life and is associated with a high mortality rate (Khoshnam et al., 2017). Dysphagia is a common morbidity of stroke, as approximately 78% of people with acute stroke have varying degrees of dysphagia (Martino et al., 2005). In addition, the occurrence of dysphagia after stroke results in various complications, such as aspiration pneumonia, malnutrition, dehydration, and even mortality, indicating that identifying effective treatments for dysphagia is critical (Cohen et al., 2016). Terré (2020) indicated that early detection and effective management of dysphagia in patients with acute stroke reduces not only the incidence of these complications but also the length of hospital stay and overall healthcare expenditures.

The white matter comprises nearly half of the volume of the brain and plays a key role in development, ageing, and many neurologic and psychiatric disorders. Thousands of myelinated fibers in the white matter allow the transfer of information, which links all brain regions into functional ensembles (Filley, 2005; Filley and Fields, 2016). Experimental evidence has demonstrated that white matter areas, including the pyramidal tract, internal capsule, corona radiata, superior longitudinal fasciculus, external capsule, and corpus callosum, are commonly implicated in swallowing control (Alvar et al., 2021). Moreover, cerebral ischaemia usually results in damage to white matter regions, especially the corpus callosum and the optic tract (Li et al., 2014a). Rodents subjected to cerebral hypoperfusion showed disintegration of the white matter tracts, as indicated by neuroinflammation, loss of oligodendrocytes, attenuation of myelin density, and structural derangement at the nodes of Ranvier (Choi et al., 2016). Patients with white matter focal vascular lesions of the white matter tend to exhibit neurobehavioral syndromes such as conduction aphasia, pure alexia, and ideomotor apraxia (Catani et al., 2012). Therefore, it is very important to investigate the white matter changes that occur in rats with dysphagia symptoms after ischemic stroke.

Once ischaemia is caused by obstruction of cerebral blood flow, angiogenesis is activated immediately in response to the loss of blood supply (Kanazawa et al., 2019). Angiogenesis, which is closely associated with factors such as vascular endothelial growth factor (VEGF), angiopoietin (Ang), and basic fibroblast growth factor (FGF2), provides not only a sufficient supply of oxygen and nutrients but also a good environment for neurons regeneration after brain injury (Sun et al., 2003; Petcu et al., 2010; Zhang et al., 2020). In recent years, with the emphasis on the neurovascular unit (NVU; which includes neurons, capillaries, astrocytes, supporting cells, and extracellular matrix), a large amount of evidence has indicated a complex link between angiogenesis and ischemic stroke (Goldman and Chen, 2011; Zhao et al., 2018). Therefore, enhancing angiogenesis may be an effective therapeutic strategy for protecting against demyelination injury and improving swallowing function after ischemic stroke.

An inflammatory response is activated in brain tissue after central nervous system injury and aggravates brain tissue injury following ischemic stroke (Cao et al., 2014). Toll-like receptor (TLR) 4, a key member of the TLR family, plays a pivotal role in the initiation of the innate immune response. TLR4 binds and interacts with downstream mitogen-activated protein kinases (MAPKs) and nuclear transcription factor-kappa B (NF-kB) to regulate inflammation by promoting the expression of interleukin (IL)-1β, IL-6, tumor necrosis factor-α (TNF-α), and other proinflammatory cytokines (Lee et al., 1999; Kyriakis and Avruch, 2001). A previous study reported that specifically inhibiting MAPK with SB203580 can reduce the increase in TNF-α, IL-1β and IL-6 expression in the hippocampus induced by mitotic factor-induced inflammation (Chaparro-Huerta et al., 2005). Studies have shown that neuroinflammation plays a key role in the pathophysiology of white matter injury in animal models of chronic cerebral hypoperfusion. Acute inflammation induced by cerebral ischaemia exacerbates tissue damage by increasing the production and release of inflammatory cytokines (Deng et al., 2014; Theus et al., 2017). Therefore, resolving inflammation is critical for protecting against brain injury after ischemic hypoperfusion.

Vagus nerve stimulation (VNS) was clinically approved by the European Commission and the US Food and Drug Administration (FDA) for the treatment of drug-resistant epilepsy and depression (Nemeroff et al., 2006; Panebianco et al., 2015). Transcutaneous auricular vagus nerve stimulation (ta-VNS) has been proven to be a novel and effective neuroprotective treatment strategy for cerebral ischaemia. A large number of studies have demonstrated that ta-VNS can reduce the infarct volume, induce angiogenesis, and improve neurological functions in a rat model of middle cerebral artery occlusion (MCAO; Jiang et al., 2016; Ma et al., 2016; Redgrave et al., 2018; Li et al., 2020b; Dawson et al., 2021). More importantly, the vagus nerve is responsible for afferent and efferent nerve signals and is closely related to swallowing movement. A systematic review reported that stimulation of the vagus nerve in cavum concha results in the activation of the nucleus tractus solitarii (NTS) and the locus coeruleus (LC; Ay et al., 2015). As the main target of VNS, the NTS, with its surrounding reticular structure and nucleus suspicion (NA), which is located in the ventral medulla oblongata, constitute the “central pattern generator” of the swallowing reflex (Broussard and Altschuler, 2000). However, the effect of ta-VNS on dysphagia symptoms after an ischemic stroke is unclear, and the exact underlying mechanisms are still undefined if ta-VNS may improve dysphagia symptoms. Therefore, in the present study, an MCAO rat model and electromyography (EMG) were used to investigate the effect of ta-VNS on dysphagia symptoms after ischemic stroke. Then, remyelination, angiogenesis, and the inflammatory response in the white matter were investigated by electron microscopy, immunohistochemistry, stereological methods, enzyme-linked immunosorbent assay (ELISA) and Western blotting. We found that ta-VNS treatment for 3 weeks improved dysphagia symptoms, increased remyelination and angiogenesis and inhibited the inflammatory response in the white matter after ischaemia.

## Material and Methods

### Animals and Experimental Design

**Figure 1:**
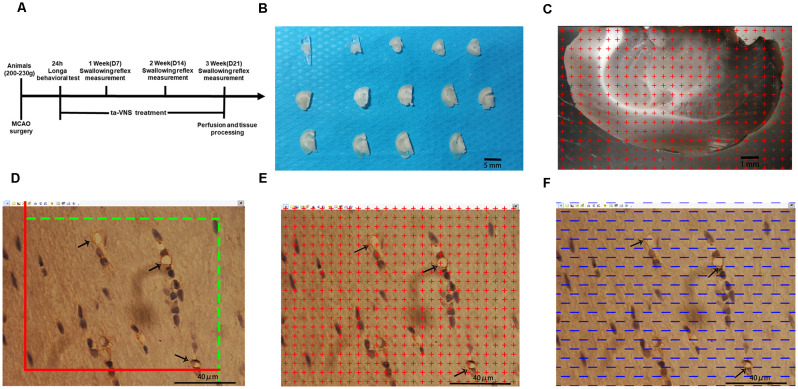
Illustrations of the methods used to quantify the white matter volume and the density of capillaries in the white matter. **(A)** Diagram of the whole experiment. **(B)** One-millimetre-thick coronal sections of the left hemisphere of rats; scale bar = 5 mm. **(C)** Illustration of the stereological counting methods used for estimating the volume of the white matter; scale bar = 1 mm. **(D–F)** Illustration of the stereological methods used for quantifying capillaries in the white matter, scale bar = 40 μm.

All animal procedures were approved by the Institutional Ethics Committee of Chongqing Medical University and performed strictly in accordance with the Guidelines for the Care and Use of Laboratory Animals. The experimental design is shown in Figure 1A. Six-week-old male Sprague–Dawley rats (200–230 g), which correspond to a weight range (6–8 weeks) frequently used for dysphagia studies on ischemic stroke, were used in the present study (Sugiyama et al., 2014; Cullins and Connor, 2019). The rats were obtained from the Experimental Animal Center of Chongqing Medical University and housed in a quiet room maintained at 21–22°C (60% humidity on a 12 h light/12 h dark cycle) and provided free access to food and water throughout the experiment. Sixty-five rats were randomly divided into three groups, i.e., the sham group (*n* = 15), control group (*n* = 25), and VNS group (*n* = 25), which received ta-VNS treatment. Changes in the swallowing reflex were measured every week (day 7 for week 1; day 14 for week 2 and day 21 for week 3). ta-VNS treatment was not given on the day of measuring the swallowing reflex, and tissues of the rats were processed after 3 weeks of ta-VNS treatment.

### Induction of Dysphagia by MCAO

We used a rat model of transient MCAO, which exhibit some symptoms of human dysphagia following an ischemic stroke in cerebral areas (Sugiyama et al., 2014). The MCAO procedure was the same as that reported previously (Belayev et al., 1996). Briefly, food intake was limited 12 h before the surgery, and the rats were anesthetized with phenobarbital sodium (40 mg/kg, intraperitoneal injection). The left common carotid artery (CCA), external carotid artery (ECA), and internal carotid artery (ICA) were carefully sequentially exposed so that the vagus and superior laryngeal nerves were kept intact. The proximal end of the ECA and CCA was ligated, and a slip knot was reserved near the bifurcation of the ICA and ECA to fix the nylon thread bolt. Nylon thread (0.32 mm; Cinontech, A5, China) was introduced into the left CCA through a small incision and gently advanced through the ICA until the black mark reached the bifurcation of the ICA and ECA. After 90 min of ischaemia, the thread was removed, and the incision was closed with sutures. Body temperature was maintained at approximately 37°C with an electrothermal pad during the experimental process. The sham rats underwent the same surgical procedures as the MCAO rats except for ligation of the CCA and ECA and introduction of the thread. Zea-Longa scores were used to evaluate the neurological deficits of the rats 24 h after MCAO, and rats with a score of 2–3 were considered successful models and were included in the follow-up experiment (Longa et al., 1989).

### ta-VNS Treatment

For ta-VNS treatment, the rats were anesthetized with 2% isoflurane, and two oppositely charged magnetic electrodes were placed inside and outside each ear over the auricular concha region. Transcutaneous electrical stimulation at a frequency of 20 Hz and an intensity of 2 mA square pulses (pulse width, 0.5 ms) was applied for a single stimulation of 30 min *via* an electrical stimulator (HANS-100, Nanjing, China). The stimulation parameters were already proven to effectively activate the vagus nerve by previous studies (Li et al., 2014b; Li S. et al., 2020). The ta-VNS procedure was administered daily at the same time between 9 a.m. and 12 a.m. to weaken the influence of biological rhythm. The animals received ta-VNS treatment daily for 3 weeks except on the days that the swallow reflex was tested. The rats in the control group and the sham group underwent the same procedure, but the stimulator was not turned on for electrical stimulation (Jiang et al., 2016).

### Measurement of the Swallowing Reflex

Rats were anesthetized with phenobarbital sodium (40 mg/kg, intraperitoneal injection) and then fixed in the supine position on a heated pad to maintain the body temperature at 37°C. A 0.5 mm catheter was inserted through the mouth, with its tip placed in the pharynx. The other end of the catheter was connected to a microsyringe pump (RWD, KDS LEGATO 130, China). Distilled water (DW) was automatically infused *via* the microsyringe pump at a flow rate of 2.0 μl/s for 20 s. The infusion was repeated three times at intervals of 3 min. Meanwhile, a midline incision was made on the ventral surface of the neck, and a pair of needle electrodes made of enamel nichrome wire was inserted into the swallowing muscles to record EMG activity. The swallowing reflex elicited by infusions of DW was assessed by EMG activity, and the number of swallows during infusion and the onset latency to the first swallow were analyzed. The number of swallows within 20 s was defined as the swallowing frequency. The onset latency to the first swallow was defined as the time required to elicit the first swallow from the onset of DW infusion. For rats in the sham group, the number of swallows and onset latency to the first swallow were measured only once throughout the whole experiment (Kajii et al., 2002).

### Perfusion and Tissue Processing

After measurement of the swallowing reflex in the 3rd week, the animals were deeply anesthetized by intraperitoneal injection of 60 mg/kg pentobarbital sodium. Then, five rats from each experimental group were randomly selected and perfused with saline followed by 4% paraformaldehyde. The cerebrum was removed from the brain and divided into two hemispheres along the sagittal suture, and the tissue was postfixed for at least 24 h after perfusion. The left hemispheres were embedded in a brain mould filled with 6% agar and cut into consecutive 1-mm-thick coronal slabs (Figure 1B). An average of 10–12 slabs were cut from the left cerebral hemispheres of each rat. Ten slabs containing white matter from the left cerebral hemisphere of each rat were randomly selected and imaged under a dissecting microscope at low magnification with a 10× objective. Then, tissue blocks approximately 1 mm^3^ in size were cut from five slabs randomly selected from each rat where the plastic sheet touched the white matter. The randomly selected blocks of white matter were subsequently placed in 10%, 20%, and 30% sucrose solutions for 1 day each. After dehydration for 3 days, the embedded samples were subjected to the isector method (CM1860, Leica) to obtain isotropic and uniform random (IUR) slices (Tang et al., 2009; Qi et al., 2020). One 4-μm-thick slice was cut from each block along the direction parallel to the IUR surface to generate sections hereafter referred to as IUR sections. The isector method ensures that capillaries of the white matter in each direction of the three-dimensional space have the same probability of being sampled.

### Immunohistochemical Staining

Briefly, frozen IUR slices were washed three times for 5 min each time in PBS (0.01 M, pH 7.4) after being warmed at room temperature. Then, the slices were immersed in acetone at 4°C for 10 min and washed three times in 0.01 M PBS. Then, the sections were incubated with 0.01 mol/L citrate buffer (pH 6.0) for 10 min at 99°C for antigen retrieval. Next, endogenous peroxidase activity in the slices was blocked with 3% hydrogen peroxide for 20 min at 37°C. After being washed three times in 0.01 M PBS for 5 min, the slices were incubated with a serum mixture (10% normal goat serum and 5% fetal bovine serum in 0.01 M PBS) for 40 min at 37°C to block nonspecific staining. Then, the slices were incubated with a mouse monoclonal anti-CD31 primary antibody (ab64543; Abcam, Cambridge, UK) diluted 1:400 in PBS at 4°C for 24 h. The slices were washed three times in PBS for 10 min each and were incubated with biotinylated goat anti-mouse immunoglobulin G secondary antibody in PBS for 2 h at 37°C. After being washed three times in PBS for 15 min each, the slices were then transferred to diaminobenzidine (DAB) solution (ZLL-9032; ZSGB, China) as a chromogen for approximately 3 min. After extensive washes with water, the cell nuclei in the slices were counterstained with Mayor’s hematoxylin (AR005; Boster Biological Engineering Co. Ltd., Wuhan, China) for 2 min. The slices were dehydrated in gradient alcohol solutions, cleared with xylene and then mounted with neutral gum.

### Stereological Analysis

#### Sampling and Image Acquisition

The slice boundary was delineated under a 4× objective lens and observed under a 100× oil lens using stereology equipment. The system randomly selected equidistant white matter areas. A total of 25 fields of view were selected from each slice, and a total of approximately 400–500 fields of view were selected for each animal. All marked blood vessels with a diameter of ≤10 μm according to a ruler were defined as capillaries (Chen et al., 2016).

#### Volume of the White Matter of the Left Hemisphere

Ten randomly selected slabs containing brain white matter from the left cerebral hemisphere of each rat were imaged under a dissecting microscope at a magnification of 10×. Then, equidistant points of light were randomly projected on every captured photograph, and the number of test points that hit the white matter was counted (Figure 1C). The total white matter volume was calculated according to Cavalieri’s principle as follows:

Vwm = t×a(p)× ΣP(wm) [Vwm represents the total volume of the white matter in the left hemisphere, a(p) is the area corresponding to each measurement point (0.59 mm^2^), t is the sample thickness (1 mm), and ΣP(wm) is the total number of test points hitting the white matter].

#### Length Density and the Total Length of Capillaries in the White Matter of the Left Hemisphere

An unbiased counting frame was randomly superimposed on every captured photograph of the 4-μm-thick slices. According to the forbidden line method, the number of cross-sections containing blood vessels with a diameter ≤10 μm (Q (cap)) was counted (Figure 1D). Capillary profiles inside the counting frame or touching the top line and the right line (inclusion lines) were included in the counts, and capillary profiles touching the left line, the bottom line, and the extensions of the right line and the left line (exclusion lines) were excluded from the counts. The length density of the capillaries in the white matter and the total length of the capillaries were calculated with the following stereological formulas (Qi et al., 2020):


Lv  (cap/wm) = ∑ Q (cap)  /  ∑A

L  (cap/wm) = Lv  (cap/wm)  /  Vwm

[ΣQ (cap) denotes the total number of capillary profiles in the white matter, and ΣA (0.0159 mm^2^) is the total area of the counting frame used for each rat].

#### Volume Density and the Total Volume of Capillaries in the White Matter of the Left Hemisphere

Equidistant points of light were randomly projected on every captured photograph of the 4-μm-thick slices. The number of test points that hit the capillaries was counted (Figure 1E). The volume density of the capillaries and the total volume of capillaries in the white matter were calculated with the following stereological formulas:


Vv  (cap/wm) = ∑ P (cap)  /  ∑P (wm)

V  (cap/wm) = Vv  (cap/wm)  × Vwm

[Vv (cap/wm) is the volume density of the capillary in the white matter, ΣP (cap) is the total number of points in the capillary section, and ΣP (wm) is the total number of points in the whole field of view].

#### Surface Area Density and the Total Surface Area of Capillaries in the White Matter of the Left Hemisphere

Test lines were randomly placed on each photograph (Figure 1F). The number of intersecting points between the test lines and the capillary luminal surface were counted. The surface area density of the capillaries and the total surface area of capillaries in the white matter were calculated with the following stereological formulas:


Sv  (cap/wm) = ∑ PI  (cap)  /  ∑L

S  (cap/wm) = SV  (cap/wm)  ×  Vwm

[ΣPI (cap) is the number of intersecting points between the test lines and the capillary luminal surface, and ΣL is the total length of all the test lines in each field of view].

### Electron Microscopy

For electron microscopy, three rats from each experimental group were randomly selected and perfused with 4% paraformaldehyde mixed with 2.5% glutaraldehyde in 0.1 M PBS. Then, the corpus callosum tissues were collected and post fixed in 4% glutaraldehyde solution for further processing. The left corpus callosum tissues were cut into 100-μm-thick slices and stained with Reynold’s lead citrate and uranyl acetate. Images were obtained at a magnification of 20,000× under a transmission microscope (TEM; Hitachi-7500, Hitachi, Ltd., Tokyo, Japan).

### ELISA

White matter tissue in the left cerebral hemisphere was rapidly removed from rats in the three groups, and a nine-fold volume of homogenate medium (0.9% normal saline) was added. Mechanical homogenization was carried out in an ice water bath to prepare 10% homogenates. The expression levels of IL-1β, TNFα, VEGF, and FGF2 were measured using rat ELISA kits (MULTI Science, China) according to the manufacturer’s protocols.

### Western Blotting

White matter tissue in the left cerebral hemisphere was collected from rats in the three groups, and protein was extracted using RIPA lysis buffer containing 1% PMSF, a phosphatase inhibitor (Beyotime Biotechnology, China). After the protein concentration was determined using a BCA kit (Beyotime Biotechnology, China), sodium dodecyl sulphate–polyacrylamide gel electrophoresis and Western blotting were carried out to measure the protein levels of TLR4, MyD88, MAPK, NF-κB, phosphorylated MAPK, and phosphorylated NF-κB.

### Statistical Analyses

All statistical analyses were performed with SPSS (version 23, IBM Corp., Armonk, NY, USA). The general condition of each rat (body weight and 24-h food/water intake), the onset latency to the first swallow and the number of swallows within 20 s were statistically analyzed using repeated measures analysis of variance (ANOVA) followed by the least significant difference (LSD) test. Electron microscopy data, quantitative stereological data, Western blot data, and ELISA data were analyzed by one-way ANOVA followed by the least LSD test. *p* < 0.05 was considered significant for all tests.

## Results

### A Rat Model of MCAO With Dysphagia Was Established, and ta-VNS Improved Dysphagia Symptoms

**Figure 2:**
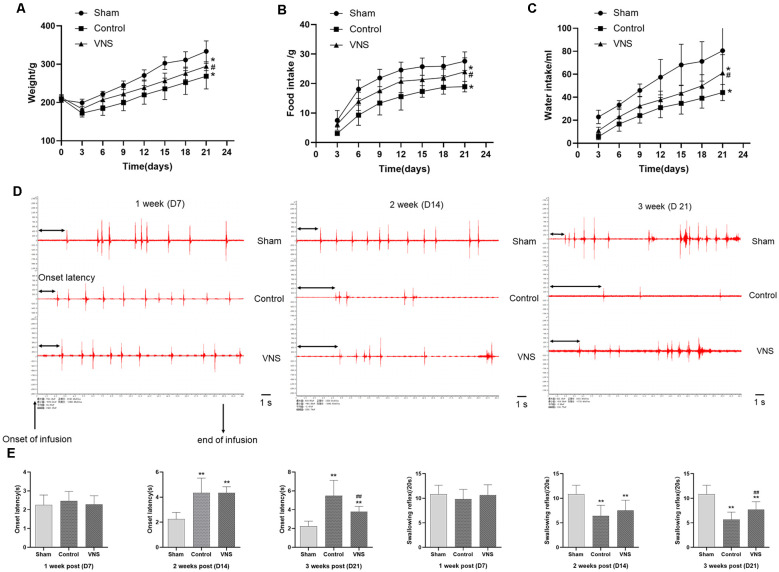
The effects of ta-VNS on dysphagia symptoms in MCAO rats. **(A–C)** Changes in the body weight and food or water intake of each group rats (mean ± SD, *n* = 10). **(D)** Representative EMG activity recorded from the swallowing muscles during swallowing elicited by infusions of DW in each group at different weeks after MCAO; scale bar = 1 s. **(E)** Changes in the onset latency to the first swallow and the number of swallows within 20 s in each group (mean ± SD, *n* = 10); *p < 0.05, **p < 0.01, the VNS group or the control group compared with the sham group; ^#^*p* < 0.05, ^##^*p* < 0.01, the VNS group compared with the control group.

To determine whether MCAO can affect swallowing behavior in rats, we measured growth, the onset latency to the first swallow and the number of swallows within 20 s. Before the experiment, there were no significant differences in body weight between the three groups. However, the bodyweight of the three groups of rats decreased sharply within 3 days after MCAO. From the 3rd day, the body weight gain of the VNS group was higher than that of the control group, and the body weights of the control group and VNS group were significantly lower than the body weights of the sham group (Figure 2A, *p* < 0.05). In addition, 24-h food intake, which was measured every 3 days, was lower in the VNS group and the control group than that in the sham group, but the 24-h food intake of the VNS group was higher than that of the control group (Figure 2B, *p* < 0.05). Furthermore, 24-h water intake, which was measured every 3 days after MCAO, was higher in the VNS group than in the control group (Figure 2C, *p* < 0.05).

EMG showed that infusion of DW elicited a swallowing reflex, and Figure 2D shows representative EMG activity recorded from the swallowing muscles during swallowing elicited by DW infusion each week after MCAO. In the 1st week, there was no significant difference in the onset latency to the first swallow or the number of swallows within 20 s elicited by infusion of DW between the three groups (Figures 2D,E, *p* > 0.05). The latency to the first swallow was significantly prolonged and the number of swallows was significantly decreased in the VNS group and the control group compared with the sham group in the 2nd week, but there was no significant difference between the VNS and the control group (Figures 2D,E, *p* > 0.05). These results seem to indicate that MCAO rats showed impairment of swallowing function and some symptoms of swallowing disorders observed in humans. In the third week, the onset latency to the first swallow was still significantly prolonged and the number of swallows was significantly decreased in the VNS group and the control group compared with the sham group. The number of swallows was significantly increased and the onset latency to the first swallow was significantly shortened in the VNS group compared to the control group (Figures 2D,E, *p* < 0.05).

### ta-VNS Induced Remyelination but Did Not Alleviate White Matter Shrinkage Induced by MCAO

**Figure 3:**
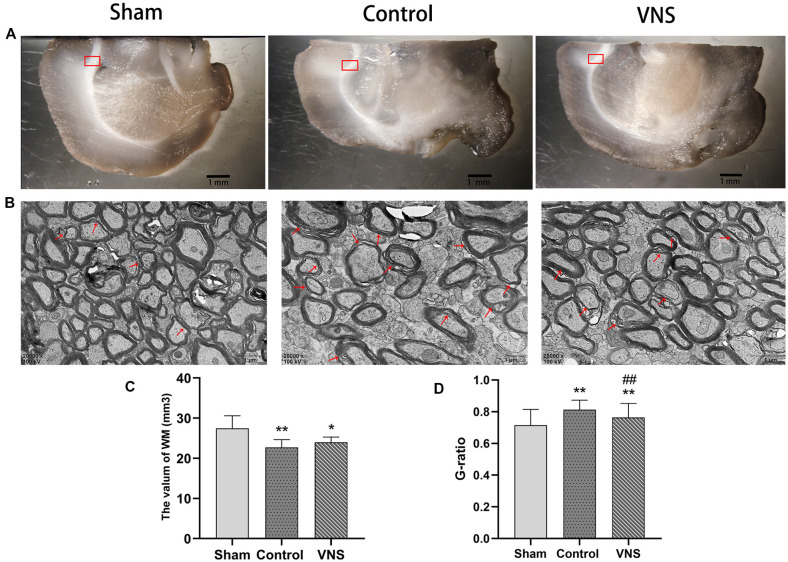
The effects of ta-VNS on the volume and remyelination of the white matter of the MCAO rats. **(A)** Representative images of white matter of rats taken under a dissecting microscope; scale bar = 1 mm. **(B)** Representative transmission electron micrographs of the left white matter in each group at a magnification of 20,000×; the red arrows indicate demyelinated axons; scale bars = 1 μm. **(C)** Total volume of the left white matter in each group (mean ± SD, *n* = 5). **(D)** Quantitative analysis of G-ratio values for all three group rats (mean ± SD, *n* = 3). *p < 0.05, **p < 0.01, the VNS group or the control group compared with the sham group; ^##^*p* < 0.01, the VNS group compared with the control group.

In the present study, stereology was used to accurately estimate the white matter volume in the three groups of rats. Figure 3A shows representative photographs containing white matter under a dissecting microscope at low magnification with a 10× objective. The white matter volume in the left hemisphere was 27.47 ± 1.40, 22.72 ± 0.86, and 22.99 ± 0.57 (mm^3^) in the sham group, the control group and the VNS group, respectively. The white matter volume in the left hemisphere in the control group and the VNS group was significantly smaller than that in the sham group (Figure 3C, *p* < 0.05), which indicated that the white matter of the brain shrank in the rats subjected to MCAO. There was no significant difference in the white matter volume in the left hemisphere between the VNS group and the control group after 3 weeks of stimulation (Figure 3C, *p* > 0.05).

Figure 3B shows representative photographs containing myelin sheathes in the corpus callosum at a magnification of 20,000× under a transmission microscope. In contrast, transmission electron microscopy of the left corpus callosum showed that the myelin sheaths were significantly thinner in the VNS group and the control group compared with the sham group, and demyelination changes were the greatest in the control group (Figure 3D, *p* < 0.01). However, the myelin sheaths in the VNS group were significantly thicker than those in the control group (Figure 3D, *p* < 0.01). Although there was no significant difference in white matter volume, the present results demonstrated that MCAO resulted in white matter demyelination and that ta-VNS treatment significantly induced remyelination in the white matter.

### ta-VNS Protected Against White Matter Damage by Inducing Angiogenesis

**Figure 4:**
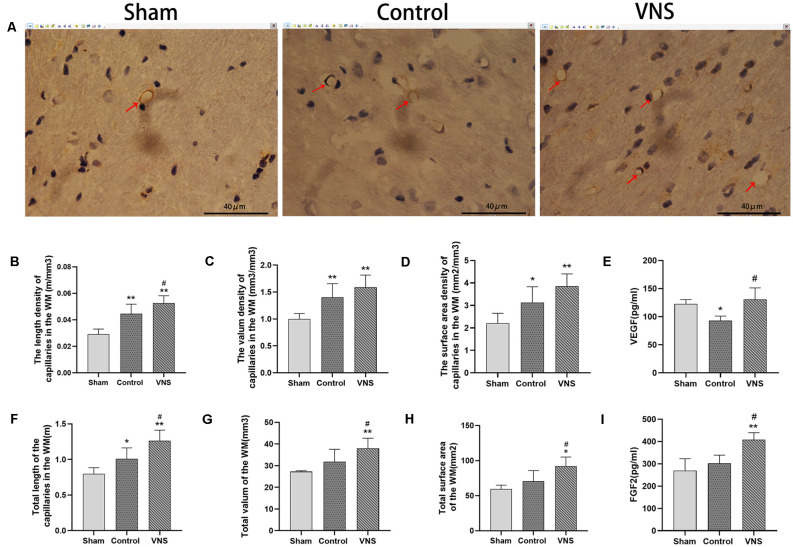
The effects of ta-VNS on angiogenesis in the white matter of MCAO rats. **(A–C)** Representative immunohistochemical staining images of white matter capillaries of rats from each group; scale bar = 40 μm/**(B–D,F–H)**. The length density (m/mm^3^), volume density (mm^3^/mm^3^), surface area density (mm^2^/mm^3^), total length (m), total volume (mm^3^) and total surface area (mm^2^) of the capillaries in the left white matter in each group (mean ± SD, *n* = 5). **(E)** The expression level of VEGF in the left white matter of rats in each group was assessed by using ELISA (mean ± SD, *n* = 3). **(I)** The expression level of FGF2 in the left white matter of rats in each group was assessed by using ELISA (mean ± SD, *n* = 3); *p < 0.05, **p < 0.01, the VNS group or the control group compared with the sham group; ^#^*p* < 0.05, the VNS group compared with the control group.

There was a significant difference in angiogenesis in the injured white matter. Immunohistochemical staining of capillaries in the left white matter of the three groups of rats is shown in Figure 4A. The length density of the capillaries in the white matter of the left hemisphere was significantly higher in the VNS group (0.053 ± 0.002; m/mm^3^) and the control group (0.045 ± 0.003; m/mm^3^) than in the sham group (0.03 ± 0.017; m/mm^3^; Figure 4B, *p* < 0.01). The length density of capillaries in the VNS group was higher than that in the control group (Figure 4B, *p* < 0.05). Similarly, the total length of the capillaries in the white matter in the VNS group (1.262 ± 0.667; m) and the control group (1.009 ± 0.686; m) was significantly longer than that in the sham group (0.797 ± 0.039; m; Figure 4F, *p* < 0.05), and the total length of capillaries in the VNS group was longer than that in the control group (Figure 4F, *p* < 0.05). The volume density of capillaries in the white matter in the VNS group (1.59 ± 0.10; mm^3^/mm^3^) and the control group (1.40 ± 0.11; mm^3^/mm^3^) was significantly higher than that in the sham group (1.00 ± 0.04; mm^3^/mm^3^; Figure 4C, *p* < 0.01), but there was no significant difference in volume density between the VNS group and the control group (Figure 4C, *p* > 0.05). The total volume of capillaries in the white matter of the left hemisphere in the VNS group (38.00 ± 2.10; mm^3^) was significantly larger than that in the control group (31.81 ± 2.58; mm^3^) and the sham group (27.25 ± 0.20; mm^3^; Figure 4G, *p* < 0.05). There was no significant difference in the total volume of the capillaries between the control group and the sham group (Figure 4G, *p* > 0.05). The surface area density of capillaries in the white matter of the left hemisphere in the VNS group (3.85 ± 0.02; mm^2^/mm^3^) and the control group (3.13 ± 0.31; mm^2^/mm^3^) was significantly higher than that in the sham group (2.21 ± 0.20; mm^2^/mm^3^; Figure 4D, *p* < 0.05). There was no significant difference in the surface area density of capillaries between the VNS group and the control group (Figure 4D, *p* > 0.05). The total surface area was significantly increased in the VNS group (92.23 ± 5.67; mm^2^) compared with the control group (70.71 ± 6.74; mm^2^; Figure 4H, *p* < 0.01), but there was no significant difference in total surface area between the control group and the sham group (58.58 ± 2.44 mm^2^; Figure 4H, *p* > 0.05).

Moreover, ELISA revealed no statistically significant differences in VEGF levels in the white matter of the left hemisphere between the VNS group and the sham group (*p* > 0.05) but a significantly higher concentration of VEGF in the VNS group than in the control group (Figure 4E, *p* < 0.05). FGF2 levels in the white matter of the left hemisphere were significantly increased both in the VNS group and the control group after 3 weeks of ta-VNS stimulation (*p* < 0.05). Compared with those in the control group, the FGF2 levels in the VNS group were significantly higher (Figure 4I, *p* > 0.05).

In summary, the present results indicate that ischemic damage induces a certain degree of angiogenesis and that ta-VNS treatment significantly promotes angiogenesis in the white matter.

### ta-VNS Alleviated the Increase in the Expression of Inflammatory Mediators and Inhibited TLR4/NF-κB Signaling and MAPK/NF-κB Signaling in the White Matter

**Figure 5:**
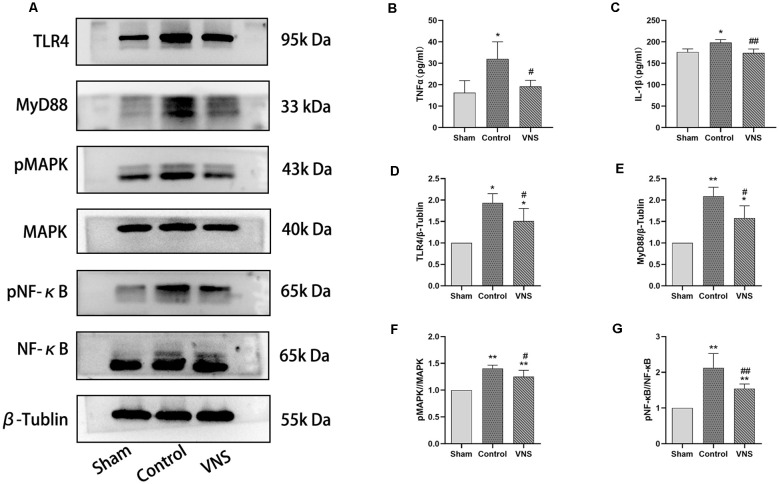
The effects of ta-VNS on the inflammatory response in the white matter of MCAO rats. **(A)** The protein expression of TLR4, MyD88, MAPK, phosphorylated MAPK, NF-κB, and phosphorylated NF-κB in the left white matter of rats in each group was measured by using Western blotting. **(B)** The expression level of TNF-α in the left white matter of rats in each group was assessed by using ELISA (mean ± SD, *n* = 3). **(C)** The expression level of IL-1β in the left white matter of rats in each group was assessed by using ELISA (mean ± SD, *n* = 3). **(D–G)** Quantitative analysis of the protein expression of these TLR4/NF-κB signaling and MAPK/NF-κB signaling in the left white matter of rats in each group (mean ± SD, *n* = 3); *p < 0.05, **p < 0.01, the VNS group or the control group compared with the sham group; ^#^*p* < 0.05, ^##^*p* < 0.01, the VNS group compared with the control group.

In the present study, we investigated the levels of TNF-α and IL-1β in the injured white matter to determine the effect of ta-VNS. Statistical analysis revealed significant group effects on the white matter levels of these two inflammatory mediators after 3 weeks of ta-VNS treatment. We found that rats in the VNS group and the control group showed significantly increased white matter levels of IL-1β and TNF-α compared with the sham group rats. The concentrations of these inflammatory mediators in the white matter of the left hemisphere were significantly lower in the VNS group than in the control group (Figures 5B,C, *p* < 0.05). These results indicate that the increase in the expression of proinflammatory markers induced by MCAO was significantly decreased by ta-VNS treatment.

According to previous studies, TLR4/NF-κB signaling and MAPK/NF-κB signaling are important for the production of proinflammatory mediators (Chaparro-Huerta et al., 2005; Theus et al., 2017). Western blotting was used to measure the expression of TLR4, MyD88, MAPK, phosphorylated MAPK, NF-κB, and phosphorylated NF-κB in the white matter of the left hemisphere. The expression levels of TLR4 and MyD88 were significantly increased in rats both in the ta-VNS group and the control group compared with the sham group rats (Figures 5A,D,E, *p* < 0.05). The TLR4 intensity and MyD88 intensity in the VNS group were significantly lower than those in the control group (Figures 5A,D,E, *p* < 0.05). Similarly, the phosphorylation levels of MAPK and NF-κB were increased in the white matter of rats in the VNS group and the control group compared to the white matter of sham group rats (Figures 5A,F,G, *p* < 0.05). The increase in the levels of phosphorylated MAPK and phosphorylated NF-κB in the white matter induced by MCAO were significantly alleviated in rats treated with ta-VNS (Figures 5A,F,G, *p* < 0.05). Our results revealed the presence of a significant inflammatory response in the injured white matter and that ta-VNS may have an anti-inflammatory effect *via* TLR4/NF-κB signaling and MAPK/NF-κB signaling.

## Discussion

Stroke-associated dysphagia is suggested to be the most important factor contributing to the risks of aspiration pneumonia, malnutrition, dehydration, and even mortality in patients (Cohen et al., 2016). In the present study, we evaluated the effects of ta-VNS on dysphagia symptoms and white matter damage, we found that ta-VNS was effective for improving the dysphagia symptoms, increasing remyelination, inducing angiogenesis, and inhibiting the inflammatory response in the white matter of cerebral ischaemia model rats.

The MCAO rat model of stroke has been widely used for decades to study the neural effects of ischaemia and has been increasingly used in recent years as an animal model to study dysphagia after stroke. Researchers found that MCAO caused a dramatic reduction in tongue protrusion (TP) and that TP values were correlated with the infarct size 7 and 24 days following MCAO (Gulyaeva et al., 2003). Impairments in licking efficiency and a compensatory increase in the number of drinking clusters were observed in MCAO rats compared with control rats (Ahmed et al., 2017). However, these studies did not use clinically relevant measures to allow translation from animal models to human studies. Cullins and Connor (2019) used video fluoroscopic swallowing studies (VFSS) to validate the unilateral transient MCAO rat model of an ischemic stroke as a translational model of poststroke dysphagia. They found clinically relevant changes in swallowing and tongue force, supporting the use of the MCAO rat model as a translational model of poststroke dysphagia (Cullins and Connor, 2019). In a recent study, Sugiyama et al. (2014) reported that an MCAO rat model can be used to study dysphagia after stroke because MCAO rats show delayed initiation of swallowing and reduced swallowing times in response to water infusion under anesthesia. Although many studies have explored the impact of different stroke locations on swallowing function, there is no common opinion in the current studies. The autonomic disturbance and hemispheric stroke difference is a recognized phenomenon, and the MCAO model involving the left hemisphere could minimize the likely autonomic disturbances, unlike the right hemisphere stroke (Oppenheimer et al., 1992; Hilz et al., 2001). Therefore, in the present study, rats subjected to MCAO involving the left hemisphere were used to model poststroke dysphagia. Consistent with previous studies, the rats subjected to MCAO exhibited swallowing deficits, and the growth of the VNS group was better than that of the control group. We observed that the number of swallows was significantly decreased and that the onset latency to the first swallow was significantly prolonged 2 weeks after MCAO. The results from the third week indicated that ta-VNS treatment relieved the symptoms of dysphagia after stroke. These results demonstrate the usefulness of the MCAO rat model for studying dysphagia after ischemic stroke, as well as the effectiveness of ta-VNS on dysphagia after an ischemic stroke.

We chose 2 mA as the stimulus parameter in the present study for ta-VNS treatment, which was not the same as those in previous invasive VNS reports. Previous studies frequently used an intensity range of 0.5 mA to 1 mA in the treatment of invasive VNS or ta-VNS. In the clinical studies of Dawson et al. (2016, 2021), patients with an ischemic stroke were implanted with a VNS stimulation device on the vagus nerve in the left carotid sheath, and then the researchers investigated the effect of 0.8 mA VNS stimulation paired with upper-limb rehabilitation on upper limb function. They found that an intensity of 0.8 mA VNS stimulation paired with rehabilitation is a novel potential treatment option for an ischemic stroke patients and has not raised safety concerns (Dawson et al., 2016, 2021). Similarly, VNS in rats which implanted with a stimulating cuff on the left cervical vagus nerve at 0.8 mA paired with chewing training significantly increased motor cortex jaw representation compared to equivalent behavioral training without stimulation (Morrison et al., 2020). Different from invasive vagus nerve stimulation, transcutaneous auricular vagus nerve stimulation effectively avoids the possible trauma that may be caused by the intervention. On the one hand, the auricular concha is the only area on the body surface that is distributed with ear vagus nerve fiber endings; on the other hand, the plasticity enhancement of VNS follows an inverted-U response of stimulation current that is influenced by pulse width, so the stimulation parameters of ta-VNS that can activate the vagus nerve have become a pivotal issue, which can directly affect the curative efficacy of the interventions (Ellrich, 2019; Liu et al., 2020). In a study by Liu et al. (2020), stimulation parameters of ta-VNS (frequency, 20 Hz; pulse width, 0.5 ms; intensity, 1.0 mA) were demonstrated to suppress epileptiform activity by activating the firing of NTS neurons (Liu et al., 2020). It has been proven that ta-VNS at 0.5 mA at 20 Hz for 1 h can promote postischemic functional recovery and angiogenesis (Jiang et al., 2016; Li et al., 2020b). However, this way of activating the vagus nerve was achieved by inserting acupuncture needles into the concha area to deliver electrical stimulation. Unlike invasive acupuncture, Li et al. (2014b) and Li S. et al. (2020) ensured the passage of the electric current by using two positive and negative electrodes placed over the skin inside and outside of the rauricular concha region, and they found that a higher intensity of 2 mA at 20 Hz can activate the vagus nerve and neuro modulation pathways (cholinergic and noradrenergic). Compared with invasive activation of the vagus nerve, it is advisable to use a higher intensity of 2 mA to activate the vagus nerve in the concha region. We finally selected an intensity of 2 mA in the present study for ta-VNS treatment and proved that ta-VNS at the parameters used in the present study exert a therapeutic effect on dysphagia symptoms.

White matter connectivity is crucial for advanced brain functions. Numerous studies have shown the presence of white matter lesions in patients with stroke, but there is limited evidence linking the white matter to swallowing control. A systematic review revealed that white matter damage can be directly tied to swallowing deficits after ischemic stroke (Alvar et al., 2021). In a clinical study, Li et al. (2014a) found that the mean fractional anisotropy of the white matter tract was significantly reduced in patients with middle artery infarction and dysphagia. A retrospective analysis suggested that white matter lesion observed on brain magnetic resonance imaging scans was an independent factor affecting various swallowing parameters; specifically, prolonged oral transit time and penetration were predicted by the severity of the white matter lesion (Moon et al., 2017). In the present study, we suspected that white matter damage is partly responsible for swallowing disorders in animals subjected to MCAO, and we used the white matter as a target for improving brain structure and function in the treatment of swallowing disorders by ta-VNS. We used electron microscopy to investigate changes in the myelin sheath in the white matter and stereological methods to accurately estimate the volume of the white matter. We found that MCAO caused significant shrinkage of the white matter. However, ta-VNS treatment for 3 weeks did not alleviate the white matter shrinkage. Interestingly, our results provided quantitative evidence that ta-VNS significantly induced remyelination in the white matter.

Both clinical and animal studies have demonstrated that VNS can promote functional recovery and exert neuroprotection against ischemic stroke (Jiang et al., 2016; Ma et al., 2016). In addition, ta-VNS paired with physical rehabilitation can improve upper limb motor function after an ischemic stroke in clinical practice (Redgrave et al., 2018; Dawson et al., 2021). However, the underlying mechanisms that mediate this beneficial therapeutic effect are unclear, and there is no unified view regarding the mechanism by which VNS treatment promotes recovery. Angiogenesis, as an important basis of neurological repair following an ischemic stroke and a novel target for stroke treatment, attracted our attention in the present study. Endogenous angiogenesis occurs 3 days after MCAO and persists for at least 21 days, resulting in improvements in functional prognosis due to increased cerebral blood flow (Sun et al., 2003). We used immunohistochemistry combined with stereology to quantitatively study capillaries in the white matter and found that ta-VNS treatment for 3 weeks induced a significant increase in angiogenesis, as indicated by an elevated capillary density. Consistent with this evidence, the white matter expression levels of VEGF and FGF2 were also significantly elevated in ta-VNS-treated rats. The results of the present study are similar to those of previous studies to some extent. For instance, a previous study revealed that ta-VNS promotes functional recovery and enhances the postischemic angiogenic response and this angiogenic response is associated with higher expression levels of brain-derived neurotrophic factor (BDNF), endothelial nitric oxide synthase (eNOS) and VEGF in the ischemic penumbra (Jiang et al., 2016). Li et al. (2020b) performed a series of experiments indicating that ta-VNS is capable of promoting angiogenesis and improving neurofunctional recovery in the chronic stage of ischemic stroke. They revealed that PPAR-γ might be involved in the promotion of angiogenesis by ta-VNS (Li et al., 2020b). However, in all previous studies, the researchers obtained indirect information from neuroimaging and molecular biology experiments and did not accurately measure capillary-related parameters. Our results accurately demonstrated that angiogenesis was promoted by ta-VNS treatment and this enhancement effect may be positively associated with VEGF and FGF2 expression in the white matter of rats subjected to MCAO by using quantitative stereology, laying a structural foundation for the improvement of dysphagia symptoms after ischemic stroke. Furthermore, the present study provided evidence that blood vessels and nerve fibers in white matter may be improved or strengthened as a whole during treated by ta-VNS stimulation after ischemic stroke.

The cerebral microvascular system plays an extremely important role in maintaining normal brain and nerve function. Enhancement of angiogenesis induced by an ischemic stroke is crucial to the modulation of neural circuits and brain function, further creating a favorable environment for neurogenesis (Li Y. et al., 2020). As an important part of the blood-brain barrier, cerebrovascular endothelial cells participate in the inflammatory response during neurovascular inflammation. The degradation of extracellular matrix and basement membrane caused by impaired endothelial function usually leads to increased vascular permeability and vascular inflammation further drives inflammation and neuronal destruction in the central nervous system after cerebral ischaemia (Frankowski et al., 2015; Ludewig et al., 2019). Researchers have found that an inflammatory environment also hinders angiogenesis. In an *in vitro* experiment, Shang et al. (2020) found that the proliferation of endothelial cells in a simulated proinflammatory microenvironment was significantly lower than that in an anti-inflammatory microenvironment and that apoptosis of endothelial cells was increased in an inflammatory microenvironment. A clinical study showed that several inflammatory mediators including TNF-α are associated with poor clinical outcomes in peripheral artery disease (Pande et al., 2015). Therefore, resolving inflammation is critical for promoting angiogenesis and protecting against brain injury after ischemic perfusion. Zhang et al. (2016) found that resolving D2 enhances postischemic angiogenesis while resolving inflammation. However, they did not provide evidence for the mechanism underlying this effect. Moreover, accumulating evidence indicates that ta-VNS causes obvious attenuation of the systemic inflammatory response evoked by endotoxin in experimental animals and that this effect is mediated by stimulation of nicotinic receptors on splenic macrophages by acetylcholine (ACh). Hence, the circuit was dubbed the “cholinergic anti-inflammatory pathway” (Hoover, 2017). Based on these results, Li et al. (2020a) found that ta-VNS treatment inhibits excessive post reperfusion inflammatory responses by enhancing α7nAChR mRNA and protein expression after brain injury. Li Y. et al. (2020) demonstrated that RenshenShouwu (RSSW) extract enhances neurogenesis and angiogenesis and this effect may be mediated by inhibition of the TLR4/NF-κB/NLRP3 inflammatory signaling pathway following an ischemic stroke in rats. In the present study, Western blot analysis revealed that the expression of proteins involved in the TLR4/NF-κB signaling pathway and MAPK signaling pathways, such as TLR4, MyD88, phosphorylated MAPK and phosphorylated NF-κB, was significantly increased in the white matter of rats subjected to MCAO. The levels of the inflammatory cytokines TNF-α and IL-1β, which are downstream of the NF-κB pathway, also increased in the left white matter of rats subjected to MCAO. Surprisingly, this increase was alleviated in rats treated with ta-VNS. Therefore, we proposed that ta-VNS might improve the inflammatory environment of the white matter by inhibiting the activation of the TLR4/NF-κB and MAPK/NF-κB signaling pathways, thus affecting endothelial cell function and promoting neurovascular unit (NVU) regeneration in the white matter and promoting swallowing function recovery after ischemic stroke.

The need for sensitive, easy-to-implement assessment methods of dysphagia symptoms after stroke is crucial in preclinical stroke research. Although VFSS is the gold standard for swallowing disorders in clinical research, it is much more difficult to do so in an awake rat, and VFSS was not sensitive enough to changes in swallowing duration in basic research under the same conditions as those used in humans (Lever et al., 2015; Cullins and Connor, 2019). EMG as an evaluation tool is widely used in the study of dysphagia after an ischemic stroke, we used the EMG signals of swallowing muscles to reflect the swallowing reflex of rats induced by DW and we found time changes in the swallowing reflex of rats in each group in the present study (Sugiyama et al., 2014; Ikeda et al., 2015). As we can see in the current cerebral ischaemia studies, VNS paired with rehabilitation training could enhance synaptic plasticity and promote recovery after stroke. It is worth noting that the assessment of the swallowing reflex in the present study may also be a form of rehabilitation training for rats with dysphagia. However, we only performed the swallowing test once a week for a total of three times, which is not enough to prove that the assessment procedure can produce a therapeutic effect compared with studies including VNS paired with up to 6 weeks of exercise training (Dawson et al., 2016, 2021; Redgrave et al., 2018). Of course, we supposed that VNS combined with swallowing training (inducing swallowing reflex to occur spontaneously by infusion of DW like the assessment method) can be considered an intervention to better explore new dysphagia treatment in future studies.

The occurrence of dysphagia after an ischemic stroke not only affects the food and water intake of patients, causes malnutrition, and affects the quality of life of patients but also increases the incidence of aspiration and pulmonary infection, which greatly increase the mortality of patients. However, treatment options for dysphagia after stroke are very limited, and swallowing is a complex, sequential movement with a fixed pattern. The relative dispersion of the swallowing center in the cortex undoubtedly increases the difficulty of dysphagia research. Identifying the key target molecules or structures for alleviating dysphagia will provide new inspiration for the clinical treatment of dysphagia. In the present study, we demonstrated that 3 weeks of ta-VNS treatment can improve dysphagia symptoms in rats subjected to MCAO. We provided evidence that ta-VNS treatment promotes angiogenesis and inhibits inflammatory response in the white matter. This finding may clarify the partial neuroprotection exerted by ta-VNS.

### Limitations

The limitations of the present study are that we did not study the effect of the intervention in the acute phase but administered ta-VNS treatment for 3 weeks beginning 24 h after MCAO. More precise intervention time points should be included in future studies. The stimulus parameters used throughout the experiment were based on previous studies, and the effects of different stimulus parameters should be compared and tested to maximize the therapeutic effect of ta-VNS. In addition, the status of the rats was also a limitation of the present study. It is known that other comorbid cardiovascular risk factors (e.g., hypertension, hyperlipidemia, diabetes, etc.) could influence prestroke autonomic disturbance and may worsen the recovery of poststroke dysphagia. From a translational research perspective, the healthy rat MCAO model cannot simulate actual clinical stroke patients. Future studies should include MCAO animal models in various disease states to elucidate the mechanisms that mediate this therapeutic effect of ta-VNS.

## Data Availability Statement

The original contributions presented in the study are included in the article, further inquiries can be directed to the corresponding author.

## Ethics Statement

The animal study was reviewed and approved by the Institutional Ethics Committee of Chongqing Medical University.

## Author Contributions

Conception and design: SW, GJ, and LY. Administrative support: SW, MF, and GJ. Collection and assembly of data: LL, QZ, MF, LZ, YQ, and YL. Data analysis and interpretation: All authors. Manuscript writing: LL and SW. All authors contributed to the article and approved the submitted version.

## Funding

This work was supported by the National Natural Science Foundation of China (NSFC, 81601967), Chongqing Health Joint Medical Research Project, Kuanren Talents Program of The Second Affiliated Hospital of Chongqing Medical University (2021MSXM144).

## Conflict of Interest

The authors declare that the research was conducted in the absence of any commercial or financial relationships that could be construed as a potential conflict of interest.

## Publisher’s Note

All claims expressed in this article are solely those of the authors and do not necessarily represent those of their affiliated organizations, or those of the publisher, the editors and the reviewers. Any product that may be evaluated in this article, or claim that may be made by its manufacturer, is not guaranteed or endorsed by the publisher.
